# Plasma exchange (PE) versus intravenous immunoglobulin (IVIG) for the treatment of Guillain-Barré syndrome (GBS) in patients with severe symptoms: A systematic review and meta-analysis

**DOI:** 10.1016/j.ensci.2023.100468

**Published:** 2023-05-25

**Authors:** Hany A. Zaki, Haris Iftikhar, Mavia Najam, Maarij Masood, Nood Dhafi R. Al-Marri, Mohamed Abdelgadir M. Elgassim, Mohamed Fayed, Eman E. Shaban

**Affiliations:** a Emergency Medicine, Hamad General Hospital, P.O. Box 3050, Doha, Qatar; b Department of Medical Education, Hamad Medical Corporation, Doha, Qatar; c Cardiology, Al Jufairi Diagnosis and Treatment, Doha, Qatar

**Keywords:** Immunoglobulins, Intravenous, Plasma exchange, Review, Systematic, Guillain-Barre syndrome

## Abstract

**Background and purpose:**

Guillain- Barré syndrome (GBS) is a neuropathic condition that leads to the rapid development of impairments and is characterized by weakness and numbness or tingling sensation in the legs and arms and sometimes loss of movement and feeling in the legs, arms, upper body, and face. Currently, the cure for the disease is yet to be developed. However, treatment options such as intravenous immunoglobulin (IVIG) and plasma exchange (PE) have been used to minimize the symptoms and duration of the disease. Therefore, this systematic review and meta-analysis compared the efficacy of IVIG and PE in treating GBS patients with severe symptoms.

**Methodology:**

Six electronic databases, including PubMed, Embase, Scopus, ScienceDirect, Medline, and Google scholar, were scoured for articles related and relevant to our research. Additionally, more studies were obtained through the reference lists of the studies retrieved from these electronic databases. Quality assessment and statistical data analysis were conducted using Review Manager software (RevMan 5.4.1).

**Results:**

The search for relevant articles resulted in 3253 articles, of which only 20 were included for review in the current study. A sub-group analysis indicated no significant difference in the curative effect (Hughes score reduces by at least one score 4 weeks after GBS treatment; OR: 1.00; 95% CI: 0.66–1.52; *p* = 1.00 and Achieving grade 0 or 1 on Hughes scale; OR: 1.03; 95% CI: 0.27–3.94; *p* = 0.97). Similarly, the statistical showed that the difference in length of hospitalization and duration of mechanical ventilation was insignificant between the IVIG and PE group (Standard Mean Difference (SMD): -0.45; 95% CI: −0.92, 0.02; I^2^ = 91%; *p* = 0.06 and SMD: -0.54; 95% CI: −1.67, 0.59; I^2^ = 93%; *p* = 0.35, respectively). Moreover, the meta-analysis did not find any significant difference in the risk of GBS relapse (RR: 0.47; 95% CI: 0.20–1.14; *p* = 0.10) and risk of complications related to the treatment regimens (RR: 1.03; 95% CI: 0.71–1.48; *p* = 0.89). However, the statistical analysis of outcomes from 3 studies showed that the risk of discontinuation was significantly lower in the IVIG group than in the PE group (RR: 0.22; 95% CI: 0.06–0.88; *p* = 0.03).

**Conclusion:**

Our study suggests that IVIG and PE have similar curative effects. Similarly, IVIG seems easier to use and thus can be preferred for treating GBS.

## Highlights

• Intravenous immunoglobulin (IVIG) and plasma exchange (PE) provide similar curative effects and relapse rates.
• The reductions in hospitalization and ventilation length, and the potential risk of complications are also similar.
• IVIG is easier to use and might be preferred for treating patients with severe symptoms of Guillain-Barré syndrome (GBS).
• Offering both treatments for patients with GBS does not influence the curative effect.
• The risk of being discontinued from IVIG treatment was significantly lower than PE treatment.

## 1 Introduction

Guillain- Barré syndrome (GBS) is a neuropathic condition that rapidly develops impairments [1,2]. It is usually characterized by weakness and numbness or tingling sensation in the legs and arms, and can be caused by respiratory infections and stomach flu. Research shows that it has a global incidence rate of 1–2 per 100,000 people yearly and affects people of all age groups [3]. However, it is slightly more common among males than females [4,5].

Currently, the cure for GBS is yet to be developed, but the standard treatments for the disease include plasma exchange (PE) and intravenous immunoglobulin (IVIG). PE was originally acknowledged as the treatment option for GBS in the mid-1980s when it was revealed to be more effective in treating GBS than conventional treatments [6]. However, research has shown that it is associated with increased risk of adverse effects and post-treatment worsening symptoms [[7], [8], [9]].

On the other hand, IVIG ameliorates the course of GBS, but its specific action mechanism is still unknown [10]. Research implies that IVIG has a similar impact as PE in treating GBS; however, it is deemed safer due to its reduced complications and risks. In contrast, Van der Meche and Schmitz [11] suggested IVIG was a more effective treatment option than PE. This study showed that at four weeks, 53% of the patients that received IVIG had improved by one or more functional grades compared to the 34% in the PE group (*p* = 0.024). A previous review article also suggested that PE may be more effective than IVIG since the relapse rates were higher in IVIG patients than in PE patients [12]. Due to these contradicting results, we conducted the current systematic review and meta-analysis to compare which treatment option between IVIG and PE was more effective in treating GBS for patients with severe symptoms.

## 2 Methodology

### 2.1 Protocol and registration

We prepared this systematic review and meta-analysis by following and observing the Cochrane collaboration guidelines and results reported per the PRISMA (Preferred Reporting Items for Systematic Review and Meta-Analyses) guidelines. The protocol was not registered in any database.

### 2.2 Literature search and eligibility criteria

Studies were identified by searching PubMed, Embase, Scopus, ScienceDirect, Medline, and Google Scholar databases using the following search terms: (Intravenous immunoglobulin) AND (plasma exchange OR plasmapheresis) AND (Guillain- Barré syndrome OR acute inflammatory demyelinating polyneuropathy OR Miller Fisher syndrome OR Acute Motor-Sensory Axonal Neuropathy OR Acute Motor Axonal Neuropathy). Afterward, two reviewers (H.Z. and H.I.) included articles that were written in English, directly compared IVIG to PE in patients with GBS or its variants, and included more than ten patients. Articles that did not meet these criteria or were animal and human model studies, systematic reviews, abstracts, letters to the editors, case reports, or evaluated acute pandysautonomia were excluded.

### 2.3 Data extraction and analysis

Two reviewers (H.Z. and H.I.) independently assessed each article and extracted the following data: Author ID (First author's last name and publication year), participants' characteristics (sample size, age and gender), dosages of the interventions, and outcomes analyzed in each study (Table 1). Discrepancies in the retrieved data were reconciled through consensus or consulting a third reviewer. Afterward, the two reviewers jointly carried out meta-analyses using the Review Manager software (RevMan 5.4.1), and classified the outcomes into primary (i.e., the curative effect of IVIG and PE) and secondary (i.e., length of hospitalization and ventilation, complications related to treatment, relapse rate, and the proportion of patients discontinued from treatment). The curative effect was evaluated through the rate of patients achieving improvement of ≥1 grades of disability scale four weeks after the treatment of GBS and the proportion of patients being able to walk unaided. Several versions of disability scales have been used; however, in this study, we considered the Hughes disability scale [13].

**Table 1:** Study characteristics.

Author ID	Participants' characteristics		IVIG administration	PE administration	Outcomes	Quality of study
	Sample, n (men)	Age				
Saad et al.2016 [14]	62 (26)	8.0 ± 4.7 years	0.4 g/kg/day for 5 days	200–250 ml/kg was administered over 7–10	Hospitalization length, mortality and curative effect	Fair
El-Bayoumi et al.,2011 [15]	41	49–143 months	0.4 g/kg/day for 5 days	One volume of PE per day	Length of PICU stay, and MV duration	Fair
Charra et al.,2014 [16]	41 (31)	37.4 ± 9.2 years	0.4 g/kg/day for 5 days	4 PE over 10–14 days	Length of ICU stay, and MV duration	Fair
Kuwabara et al.,2001 [17]	24 (11)	NR	400 mg/kg/day for 5 days	50 ml/kg plasma was treated 4–8 times daily.	Curative effect	Fair
Elahi et al.,2019 [18]	78 (47)	6.64 ± 3.06 years	0.4 g/kg/day for 5 days	One-volume PE per day	Length of PICU stay, and MV duration	Poor
Tsai et al.,2007 [19]	24 (19)	7–79 years	0.4 mg/kg for 5 days	40–50 ml/kg, 4–8 times	Hospitalization length, and Complications	Fair
Bondi et al.,2021 [20]	70 (38)	NR	NR	NR	Length of ICU stay	Poor
Beydoun et al.,2020 [21]	6586 (3711)	NR	NR	NR	Hospitalization length, and mortality and	Poor
Ye et al., 2015 [22]	64 (40)	NR	0·4 g/kg for 5 days	Five 50 ml/kg PEs	Curative effect	Fair
Rodprasert.,2014 [23]	40 (23)	NR	0.4 g/kg/day for 5 days	200–250 ml/kg of plasma in 3–5 sessions within 7 to 14 days	Curative effect	Poor
Anwer et al.,2020 [24]	34 (21)	9 ± 3 years	NR	NR	Curative effect, and mortality	Fair
Ashalatha.,2002 [25]	97 (61)	NR	0.4 g/kg/day for 5 days	100 ml/kg for 5 sessions within 7–14 days	Curative effect, MV duration and length of hospitalization	Poor
Mallick et al.,2019 [26]	49 (32)	37.4 ± 9.2 years	0.4 g/kg/day	4 PE for 10 days every alternate day	Length of ICU stay, MV duration and survival rate	Poor
Ravasio et al.,1995 [27]	50 (36)	54.42 ± 20.25 years	NR	Mean PE was 1900 ml/session for 2–15 sessions	Curative effect	Fair
Bril et al.,1996 [28]	50 (26)	44.4 ± 2.6 years	0.5 g/kg for consecutive days up to a total dose of 2.0 g/kg	200–250 ml/kg plasma volume for 5 sessions within 7–10 days	Curative effect	Poor
Diener et al.,2001 [29]	57	NR	0.4 g/kg/day for 5 consecutive days	5 treatments of PE	Curative effect and complications	Poor
Cecil et al.,2014 [30]	50	NR	0.4 g/kg/day for 5 days	5 cycles of 50 ml/kg PE over 8–13 days.	Curative effect	Fair
PSGBS trial group.,1997 [31]	379 (225)	NR	0.4 g/kg/day for 5 days	Five 50 ml/kg PE within 8–13 days.	Length of hospitalization, Curative effect and relapse	Good
Romano et al.,1998 [32]	54 (26)	NR	NR	NR	Relapses	Fair
Van der Meche et al.,1992 [11]	147	47.5 ± 19.2 years	0.4 g/kg/day for 5 subsequent days	200–250 ml/kg of PE for 5 sessions within 7–14 days.	Curative effect and complications	Fair

Note: IVIG; intravenous immunoglobulin: PE; Plasma Exchange: MV; Mechanical ventilation: ICU; intensive care unit: PICU; Pediatric intensive care unit: TPE: Therapeutic plasma exchange: NR; Not Reported*.*

All outcomes except length of hospitalization and ventilation were binary, therefore, the simple Odds ratio (OR) was used for the calculations of the overall curative effect, while the risk ratio (RR) was used for the other binary outcomes. Conversely, standard mean difference (SMD) was used for the continuous data. Heterogeneity was also calculated using the I^2^ statistics, of which values ranging between 0 and 50%, 51–70%, and above 70% were considered low, moderate, and high, respectively.

### 2.4 Quality assessment

The quality assessment was performed using the Cochrane's risk of bias tool provided in the Review Manager software (RevMan version 5.4.1), and was based on the following elements; selection, performance, attrition, and reporting bias. Using these elements and the Agency of Healthcare Research and Quality standards (AHRQ) standards, the overall quality of each study was evaluated (Table 1). According to these standards, good quality was assigned when all the assessment criteria were met while poor quality was assigned when two or more assessment criteria were of high risk of bias. Finally, fair quality was assigned when only one criterion was not met or two criteria had unclear risk of bias.

## 3 Results

### 3.1 Search results

**Fig. 1:**
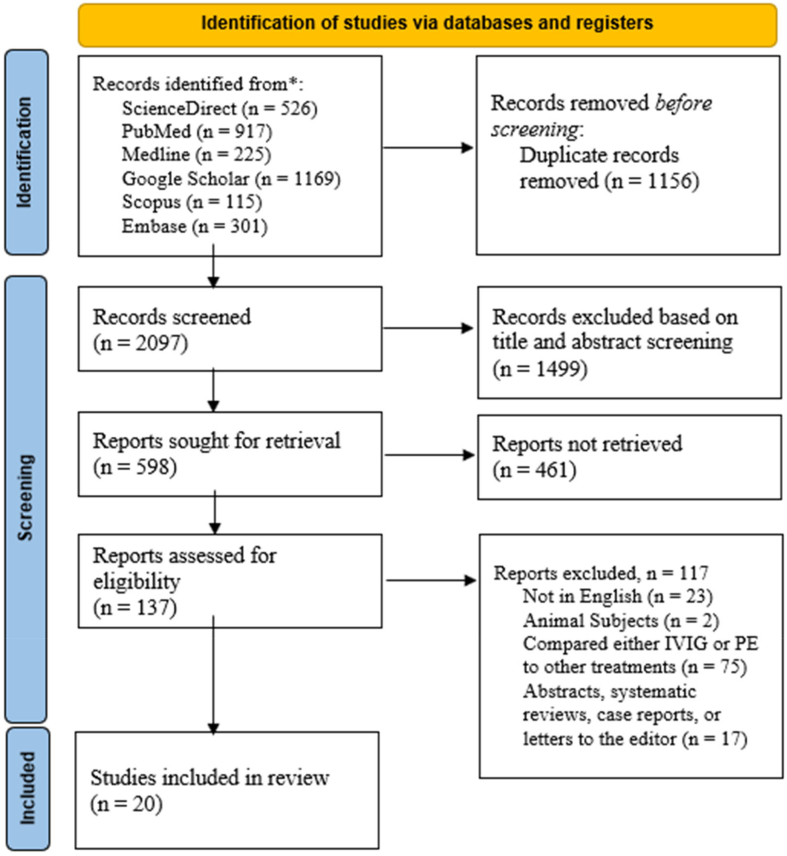
Flowchart of the systematic literature search on six electronic databases according to the PRISMA guidelines. The flow chart also summarizes the screening and exclusion and inclusion procedure.

The detailed search through the electronic databases outlined earlier resulted in 3253 articles. After analyzing these articles, we identified 1156 duplicate articles, which were excluded from the review. The remaining 2097 articles had their titles and abstracts screened, of which only 598 met the screening criteria. Of the 598 articles, 461 were not retrieved, and the other 137 were assessed using the eligibility criteria. This assessment led to the inclusion of only 20 articles while the other articles were excluded as follows; 23 were published in languages other than English, 17 were designed as either systematic reviews and meta-analyses, abstracts without full articles, letters to the editor or case reports, 2 included either animal subjects or human models, and 75 compared either IVIG or PE to other treatment options. The selection results are shown using the PRISMA flow diagram (Fig. 1).

### 3.2 Primary outcome: curative effect

**Fig. 2:**
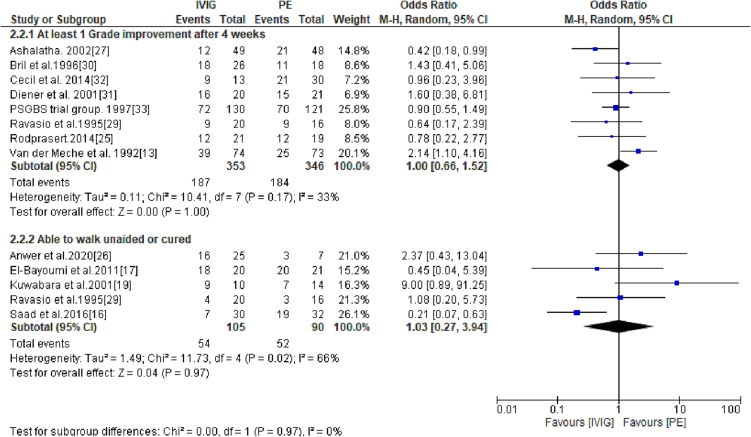
A forest plot showing subgroup analysis of patients achieving at least 1 score reduction in Hughes scale 4 weeks after GBS treatment and those who were cured (achieved grade 1 on Hughes scale).

The curative effect of IVIG and PE was assessed by identifying the number of patients that had achieved at least 1 score reduction in the Hughes scale 4 weeks after GBS treatment or those who were cured (grade 1 on the Hughes scale) or those who were able to walk without support (grade 2 on the Hughes disability scale). The pooled results showed that odds of achieving at least one grade disability improvement was similar for patients treated with IVIG and PE (OR: 1.00; 95% CI: 0.66–1.52; *p* = 1.00) (Fig. 2). Similarly, there was no difference in the number of patients being cured (grade 0 or 1 on Hughes scale) after treatment with either PE or IVIG (OR: 1.03; 95% CI: 0.27–3.94; *p* = 0.97) (Fig. 2).

### 3.3 Secondary outcomes

#### 3.3.1 Hospitalization and ventilation duration

9 of the 20 studies evaluated the duration patients treated with either IVIG and PE spent in the hospital or intensive care units (ICUs). A meta-analysis of outcomes from these studies showed that patients that received IVIG spent less time in hospitals and ICUs; however, a comparison with PE shows that the difference was statistically insignificant (SMD: -0.45; 95% CI: −0.92, 0.02; I^2^ = 91%; *p* = 0.06) (Table 2). Additionally, analysis of outcomes from 5 studies showed that mechanical ventilation (MV) duration was similar in both IVIG and PE groups (SMD: -0.54; 95% CI: −1.67, 0.59; I^2^ = 93%; *p* = 0.35) (Table 2).

**Table 2:** Meta-analysis results of secondary outcomes.

Outcomes	Studies analyzed	Effect size (95% CI)	*P*-value	I^2^
Length of Hospitalization	9	SMD: −0.45 (0.92–0.02)	0.06	91%
MV Duration	5	SMD: −0.54 (−1.67–0.59)	0.35	93%
Complications related to treatment	4	RR: 1.03 (0.71–1.48)	0.99	0%
Discontinuation	3	RR: 0.22 (0.06–0.88)	0.03	19%
Relapse	3	RR: 0.47 (0.20–1.14)	0.10	0%

#### 3.3.2 Complications related to treatment, discontinuation and relapse rate

4 of the 20 studies evaluated complications related to treatment as one of their outcomes. The pooled data from these studies showed that the risk of patients experiencing complications after treatment with PE and IVIG was statistically insignificant (RR: 1.03; 95% CI: 0.71–1.48; *p* = 0.89) (Table 2). The results also show that the frequency of relapse tends to be lower when using IVIG as opposed to PE (7/173 (4%) vs. 16/176 (9%), respectively). However, the pooled results show that risk of GBS relapse after treatment with either PE or IVIG was not statistically different (RR: 0.47; 95% CI: 0.20–1.14; *p* = 0.10) (Table 2). On the other hand, our meta-analysis shows that the risk of being discontinued from IVIG treatment was significantly lower compared to PE treatment (RR: 0.22; 95% CI: 0.06–0.88; *p* = 0.03) (Table 2).

#### 3.3.3 Publication bias

**Fig. 3:**
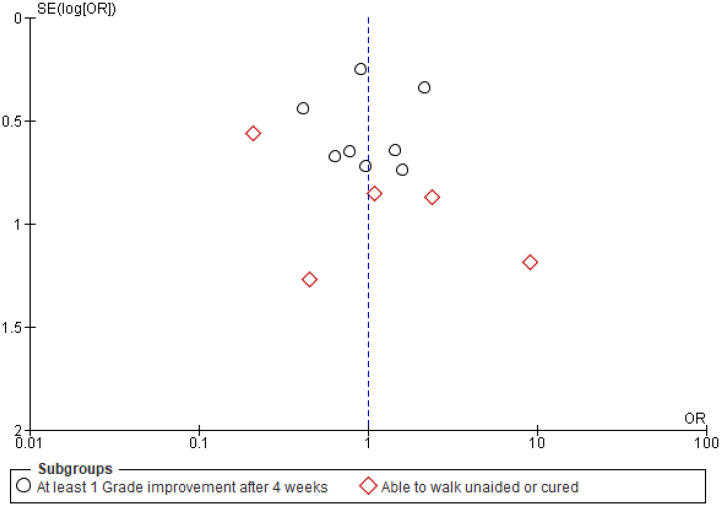
Funnel plot showing that there was no publication bias for curative effect outcomes since it was symmetrical.

Using a funnel plot, we were able to analyze the publication bias of the main outcomes. Based on the visual inspection, we observed that the funnel plot was symmetrical meaning that there was no publication bias for the main endpoint (Fig. 3).

## 4 Discussion

The cure for GBS is yet to be developed; however, treatment options such as PE and IVIG have been developed to manage the symptoms and reduce the severity of the disease. The results of our study have suggested that IVIG and PE have a similar effect on the reduction of hospitalization and ventilation durations and similar curative effects for patients with GBS.

### 4.1 Curative effect of IVIG versus PE

Our study has reported an insignificant difference in the curative effect of IVIG and PE. This finding is further supported by a previous meta-analysis of 5 randomized trials, which reported that IVIG and PE showed a similar effect on the improvement of disability scores (RR: -0.02; 95%CI: −0.25, 0.20; *p* = 0.83) [33]. Even though we used 4 weeks after treatment as the cut-off for the analysis of disability scores, evidence shows that the improvement in disability scores can be observed as early as 2 weeks after treatment. For instance, Ye and colleagues reported that at 2 weeks of follow-up PE was a more effective treatment than IVIG (*p* = 0.03) [22]. This significant change was attributed to the fact that PE significantly improved the nerve functions defects in patients with GBS. However, the results of that study were affected by the small sample bias.

### 4.2 Effect of PE versus IVIG on hospitalization and ventilation duration

Our meta-analysis has also shown that the hospitalization duration of GBS patients tends to be shorter when IVIG is used as the treatment option for GBS; however, the difference compared with PE is not statistically different. Conversely, evidence in some studies has shown that patients receiving IVIG treatment can have significantly shorter hospital stays [16,18]. This short hospital stay can be explained by the fact that IVIG also considerably reduces the time to attain better functional grades for patients with GBS [11,34].

Moreover, we found that the time until discontinuation from MV was similar in both IVIG and PE groups. The finding is contradicted by Mallick and colleagues, who reported that the patients treated with IVIG were weaned from MV earlier than the patients treated with PE (*p* = 0.002) [26]. However, the results of that study cannot be used to guide clinical treatment with IVIG since the study included a small number of patients and was designed as an observational study with poor methodological quality.

### 4.3 Safety, relapse rate and ease of use

Safety is an important outcome that needs to be evaluated when comparing the efficacy of any treatment measures. Our analysis did not demonstrate any significant difference in complication rates between patients receiving IVIG and PE. However, one study which led to significant differences in the analysis was eliminated because we could not tell whether these complications were a result of the mechanical ventilation or the treatments used for GBS [26].

It is also important to note that cured patients (Grade 1 on the GBS disability scale) or those having improved functional grade scores (≥1 improvement in the GBS disability scale) after treatment with IVIG and PE can have a relapse of GBS. Our meta-analysis shows that even though the difference between PE and IVIG is not statistically different, relapses were more frequently associated with PE treatment than IVIG (4% (7/173) vs. 9% (16/176). However, a previous review article appeared to unexpectedly report high relapse rates among GBS patients treated using IVIG (13% (17/126) and 6.2% (19/ 306), for IVIG and PE, respectively) [12]. The difference in that review can be attributed to the fact that it did not include studies that directly compared IVIG to PE.

Additionally, evidence from our analysis seems to support that IVIG is easier to use than PE since significantly less patients were discontinued from the IVIG treatment than PE treatment. These results are also in line with the findings of a previous review article that reported the risk of discontinuation from treatment was 0.14 times less likely to be observed in IVIG-treated patients than PE-treated patients [33]. In addition, IVIG can be considered easier to administer since IVIG does not require any special equipment or specially trained staff and only requires access to a single peripheral vein treated with IVIG compared to PE, which requires inserting a central venous line, a PE machine, and highly skilled personnel.

### 4.4 Limitations of our study

The current study had several limitations, including high heterogeneity in the analysis of the primary outcomes, especially hospitalization and ventilation duration. This heterogeneity was expected since the review included studies with varying sample sizes. The review also included a nationwide study of patients receiving different IVIG dosages, thus contributing to the high heterogeneity. The fact that volumes of PE varied from study to study, it is possible that this variation was a significant contribution to the heterogeneity. However, the heterogeneity did not influence the results of our meta-analyses since most of the studies had fair to good methodological quality, meaning that the publication bias was minimized. The other limitation was based on the eligibility criteria, which allowed the inclusion of studies published in English only. This criterion might have led to the omission of studies relevant to our study that could have been used to enhance our scientific research and improve the statistical power of our meta-analyses. Moreover, most of the studies included in this review did not evaluate some important variables, such as mortality rates; hence it is impossible to outline the effect of the treatment regimens on the mortality rate of GBS patients.

## 5 Conclusion

The current study suggests that IVIG and PE have similar curative effects, relapse rates, reductions in hospitalization and ventilation length, and the potential risk of complications. However, the analysis suggests that IVIG is easier to use. Based on these results, we can recommend that IVIG be preferred for treating patients with severe symptoms of GBS. However, this recommendation is offered with some caution. First, it should be noted that GBS still causes a substantial number of deaths; thus, the simplicity of the IVIG should not tempt health centers without sufficient and appropriate intensive care facilities to care for patients with GBS. Additionally, the effect of IVIG and albumin on patients with GBS remains uncertain. The evidence has also shown that offering both treatments for patients with GBS does not influence the curative effect; thus, health professionals should decide which treatment is most suitable for their patients.

## Authorship declaration

All authors are in agreement with the content of the manuscript.

## CRediT authorship contribution statement

**Hany A. Zaki:** Conceptualization, Data curation, Formal analysis, Methodology, Writing – original draft. **Haris Iftikhar:** Conceptualization, Data curation, Funding acquisition, Methodology. **Mavia Najam:** Conceptualization, Data curation, Methodology. **Maarij Masood:** Supervision, Validation, Writing – review & editing. **Nood Dhafi R. Al-Marri:** Validation, Writing – review & editing. **Mohamed Abdelgadir M. Elgassim:** Validation, Writing – review & editing. **Mohamed Fayed:** Supervision, Validation, Writing – review & editing. **Eman E. Shaban:** Conceptualization, Data curation, Formal analysis, Supervision, Writing – original draft.

## Declaration of Competing Interest

The authors declare that they have no known competing financial interests or personal relationships that could have appeared to influence the work reported in this paper.
